# Association between the ossific nucleus and osteonecrosis in treating developmental dysplasia of the Hip: updated meta-analysis

**DOI:** 10.1186/s12891-017-1468-6

**Published:** 2017-04-20

**Authors:** Rafal Niziol, Michael Elvey, Evangelia Protopapa, Andreas Roposch

**Affiliations:** Great Ormond Street Hospital for Children and UCL Institute of Child Health, 30 Guildford Street, London, WC1N 3EH UK

## Abstract

**Background:**

A meta-analysis concluded that there was no effect of the femoral head ossification and the incidence of osteonecrosis in the treatment of developmental dysplasia of the hip (DDH), unless only osteonecrosis grades II-IV were considered. The meta-analysis, limited due to the small number of studies available at that time, identified a need for an update as further research emerges. We observed a trend in recent years towards delaying treatment of DDH in the absence of an ossified nucleus. Numerous new publications on this topic encouraged us to update the 2009 meta-analysis.

**Methods:**

We performed a systematic review of the literature from 1967 to 2016 and included studies that reported on the treatment of DDH, the ossific nucleus and osteonecrosis. Two independent reviewers evaluated all articles. We performed a meta-analysis with the main outcome defined as the development of osteonecrosis of the femoral head at least two years after closed or open reduction.

**Results:**

Of four prospective and ten retrospective studies included in the systematic review, 11 studies (1,021 hips) met the inclusion criteria for the meta-analysis. There was no significant effect of the ossific nucleus on the development of all grades of osteonecrosis (relative risk, 0.88; 95% confidence interval, 0.56–1.41) or osteonecrosis grades II–IV (0.67; 0.41–1.08). In closed reductions, the ossific nucleus halved the risk for developing osteonecrosis grades II–IV (0.50; 0.26–0.94).

**Conclusions:**

Based on current evidence there does not appear to be a protective effect of the ossific nucleus on the development of osteonecrosis. In contrast to the previous meta-analysis, this update demonstrates that this remains the case irrespective of the grade of osteonecrosis considered relevant. This updated meta-analysis is based on twice as many studies with a higher quality of evidence.

**Electronic supplementary material:**

The online version of this article (doi:10.1186/s12891-017-1468-6) contains supplementary material, which is available to authorized users.

## Background

Some surgeons believe that in the treatment of developmental dysplasia of the hip (DDH), osteonecrosis may be avoided by intentionally delaying a closed or open reduction until the appearance of the ossific nucleus [1–3]. Results of published studies remain inconsistent with some authors advocating a protective effect of the ossific nucleus [1, 3–5] and others demonstrating no effect [6–8].

A previous meta-analysis of six observational studies [9] concluded that the presence of the ossific nucleus at the time of hip reduction had a protective effect against the development of grade II-IV osteonecrosis according to Bucholz and Ogden [10] or Kalamchi and MacEwen [11]. However, this effect was lost when osteonecrosis of any grade was considered. It also showed that in closed reductions an ossified nucleus reduced the risk of osteonecrosis by 60%, whereas no effect was seen in open reductions. Due to the moderate quality of evidence, the meta-analysis identified a need for further research [9]. With an increase in the number of studies seeking to clarify the effect of the ossific nucleus [1–3, 5–8, 12–14] we sought to update the meta-analysis.

This study aimed (i) to determine the effect of the presence of the ossific nucleus on the development of osteonecrosis and (ii) to assess whether the type of reduction performed or the grade of osteonecrosis considered relevant would affect the conclusion.

## Methods

### Search strategy

We updated a previous (1960–2007) systematic review with an electronic search of the literature for the period of May 2007 to November 2016. We identified articles reporting on any association between the ossific nucleus and osteonecrosis. In line with the PRISMA (Preferred Reporting Items for Systematic Reviews and Meta-Analyses) statement [15], we included MEDLINE and EMBASE databases and combined MeSH (Medical Subject Headings) and EMBASE terms and free text words in Dialog Data Star® including the terms *ossific, nucleus, hip* and *dislocation.* We also searched the DARE database and Cochrane Library.

Two reviewers (AR, RN) independently screened titles and abstracts of eligible citations and determined if they met the inclusion criteria. Selected articles were evaluated independently and disagreements resolved in consensus. In this process both reviewers demonstrated substantial [6] agreement (kappa = 0.72).

### Inclusion and exclusion criteria

This systematic review included studies of any design reporting on (i) the presence or absence of the ossific nucleus of the proximal femoral epiphysis on pre-reduction radiographs or ultrasound and (ii) osteonecrosis as an outcome of the treatment of DDH in children up to 18 years. We included studies which defined osteonecrosis by radiographic criteria (Bucholz and Ogden [10] or Kalamchi and MacEwen [11]). We excluded studies with a follow-up of less than two years and studies reporting on neuromuscular hip disorders, teratological hip dislocation and septic arthritis [2, 4, 5, 7, 13, 14, 16]. We excluded paper written in languages other than English, Polish and German.

### Data extraction and outcome measures

Two reviewers (AR, RN) independently extracted all data relevant for systematic review and meta-analysis with use of a data collection form [9], ensuring precise collection of all relevant information. We resolved disagreements in consensus.

We assessed the quality of evidence using the four domains of the GRADE (Grading of Recommendations Assessment, Development and Evaluation) statement [17]: *study design*, *study quality, consistency* and *directness*. We used clinical homogeneity as a criterion for pooling data between studies. We defined clinically homogeneous studies as those with comparable populations, interventions and outcomes measured at a similar time point. We also tested for statistical homogeneity as described below.

### Statistical analysis

We quantified agreement between reviewers with the simple kappa statistic [18] and reported treatment effects as relative risks. We used the Q-test at the 10% significance level to test for statistical homogeneity and Higgins’ I^2^ statistic to determine the percentage of total variation across studies due to heterogeneity [18]. RevMan 5.3 software was used to perform the meta-analysis. We employed fixed effects model in cases of statistical homogeneity (I^2^ < 50%) and random-effect models if there was statistical heterogeneity. However, we also performed additional fixed and random effects models on all groups analysed for data comparison. We performed subgroup analyses based on grade of osteonecrosis and based on closed and open reductions. Funnel plots used to test publication bias were generated using the RevMan 5.3 software.

## Results

### Search and selection

**Fig. 1:**
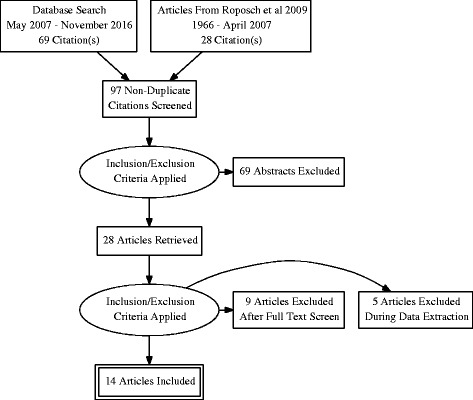
Flow diagram showing the search and selection process

The electronic search revealed 97 studies (Fig. 1), of which 69 studies did not meet the inclusion criteria as per abstract review, resulting in 28 articles being evaluated. We excluded another 14 studies: four studies failed to comment on the ossific nucleus, five studies reported on only patients with ossific nucleus or with osteonecrosis, five studies failed to present numbers for hips with ossific nucleus and osteonecrosis. In total, we included 14 studies in this systematic review. Three studies commented on the association between ossific nucleus and osteonecrosis but could not be included in the meta-analysis as they lacked detailed data [5, 13] or used a case-control design [3].

The prevalence of osteonecrosis ranged from 6 to 48% across the 14 studies. Nine studies (64%) [4, 6–8, 12–14, 16, 19] showed no relationship between the ossific nucleus and osteonecrosis; four studies (29%) [1–3, 5] claimed a protective effect of the ossific nucleus and one study (7%) [20] did not comment on a potential relationship.

### Quality of evidence

We identified one prospective cohort study [12], eleven retrospective cohort studies [1–4, 7, 8, 13, 14, 16, 19, 20], and two case series [5, 6]. Eight studies (57%) [1–3, 7, 8, 14, 16, 20] directly investigated the relationship between the presence of the ossific nucleus and osteonecrosis. The remaining six studies (43%) [4–6, 12, 13, 19] reported on this relationship without it being the primary objective of the research. Seven studies (50%) [6, 8, 12–14, 19, 20] reported details about the sampling frame. The remaining studies did not comment on sampling. One study (7%) [20] analyzed a single-surgeon’s series, seven studies (50%) [5, 7, 8, 12, 13, 16, 19] included patients from two or more surgeons of the same institution; the number of surgeons involved in the treatment of included cases of DDH remained unclear in six studies (43%) [1–3, 6, 14, 16].

We found consistency among all studies in that neuromuscular hip disease, teratological dislocation and cases with missing imaging were not included. Other exclusion criteria were failed surgical treatment prior to admission [2, 4, 5, 7, 13, 14, 16] and postoperative septic arthritis [13, 19]. There was inconsistency with regard to the age threshold below which patients were excluded from the research, namely no threshold [1, 3, 5–7, 12–14, 16], one month [8], two months [20], three months [4, 11] and six months [19]. Four studies (29%) limited the upper age limit of patients included in the research to 18 months [8, 14], 20 months [20] or 24 months [4].

The number of participants in single studies varied from 23 to 148, the average number being 67. There were 166 cases of bilateral DDH (range, 0–38). The mean age at hip reduction was 8.6 months (range, 0.7–35). Four studies (29%) included patients treated by closed reduction only, with a mean age at reduction of 8.9 months (range, 0.8-35) [1, 14, 16, 20]. Four studies (29%) included patients treated by open reduction only, with a mean age at reduction of 11.1 months (range, 2.4 – 24) [3–5, 12]. The remaining six studies (43%) included patients undergoing either closed or open reduction, with no age threshold stated for the preferred method of treatment [2, 3, 7, 8, 13, 19].

Six studies (43%) [1, 7, 8, 12, 14, 16] presented the relationship between the presence or absence of the ossific nucleus and osteonecrosis in a two-by-two table and we were able to extract this data from another five studies (36%) [2, 4, 6, 19, 20]. Three studies (22%) commented on statistical power in that they had 80% power to detect differences of 20% [7], 24% [8] and 30% [13] in the prevalence of osteonecrosis between patients with and without an ossific nucleus (Table 1).

**Table 1:** Ranking of studies based on their methodological quality according to the GRADE Statement [17]

Domains of GRADE →	Study design	Study quality						Directness		Consistency
Study	Was it a cohort study?	Did the study directly compare the relationship of ON and osteonecrosis?	Was radiographic followup at least 4 years in all included cases?	Was loss-to-followup less than 20%?	Were outcomes ascertained blinded to outcome?	Were multivariate analyses performed?	Was a risk ratio with confidence intervals reported?	Were all conclusions about the relationship between ON and osteonecrosis supported by data?	Were participants, interventions and outcome measures similar to those of interest in clinical practice?	Were estimates of effect (results) similar to other studies?
Roposch et al. (2011) [8]	✓	✓	✓	✓	✓	✓	✓	✓	✓	✓
Sllamniku et al. (2013) [14]	✓	✓	✓	✓	✘	✘	✓	✓	✓	✓
Sibinski et al. (2009) [16]	✓	✓	✓	✓	✘	✘	✘	✓	✓	✓
Pospischill et al. (2011)	✓	✘	✓	✘	✘	✓	✓	✓	✓	✓
Madhu et al. (2013) [19]	✓	✘	✘	✓	✓	✘	✘	✓	✓	✓
Luhmann et al. (1998) [7]	✓	✓	✘	✓	✘	✘	✘	✓	✓	✓
Segal et al. (1999) [3]	✘	✓	✘	✓	✘	✓	✓	✓	✓	✘
Clarke et al. (2005) [2]	✓	✓	✘	✓	✘	✘	✘	✓	✓	✘
Carney et al. (2004) [1]	✓	✓	✘	✓	✘	✘	✘	✓	✓	✘
Cooke et al. (2010) [20]	✘	✓	✓	✓	✘	✘	✘	✘	✓	✘
Agus et al. (2002) [12]	✘	✘	✘	✓	✘	✘	✘	✓	✓	✓
Konigsberg et al. (2003) [6]	✓	✘	✘	✓	✘	✘	✘	✘	✓	✓
Tarassoli et al.(2014) [4]	✓	✘	✘	✓	✘	✘	✘	✘	✓	✘
Zamzam et al. (2009) [5]	✓	✘	✘	✓	✘	✘	✘	✘	✓	✘

### Confounders and effect modifiers

All studies identified age at reduction as a potential confounder; however only ten studies (72%) [1–3, 5–8, 12–14] provided supportive statistical analyses. Two studies (14%) [6, 14] found that hips reduced after the age of ten months were more likely to develop osteonecrosis.

Carney et al. [1] found an increased risk of osteonecrosis when an adductor tenotomy was omitted during a closed reduction (*p* = 0.007). Pospischill et al. [13] found an increased risk for osteonecrosis if a concomitant osteotomy was done (*p* < 0.05).

The use of harness treatment, preoperative traction and adductor tenotomy was not universal across all studies. In ten studies (72%) [1–3, 6–8, 13, 14, 16, 20] preoperative traction was employed. However, only five studies (36%) [1, 3, 7, 8, 13] provided statistical analyses and found no relationship between traction and osteonecrosis.

Five studies (36%) [1, 3, 7, 8, 13] examined the effect of failed harness treatment and osteonecrosis and no such effect was seen.

### Meta-analysis

**Fig. 2:**
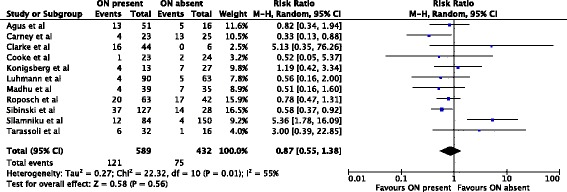
Forest plot for the development of osteonecrosis of any grade at two or more years following open or closed reduction. Including the data of 11 studies, 1,021 hips were analyzed. There was no statistically significant association between the ossific nucleus and the development of osteonecrosis

A meta-analysis of 11 studies (1,021 hips) [1, 2, 4, 6–8, 12, 14, 16, 19, 20] showed no protective effect of the ossific nucleus on the development of osteonecrosis grades I-IV. 121/589 (21%) hips with an ossified nucleus developed osteonecrosis compared with 75/432 (17%) in the group without an ossific nucleus (relative risk, 0.87; 95% confidence interval, 0.55-1.38) (Fig. 2).

**Fig. 3:**
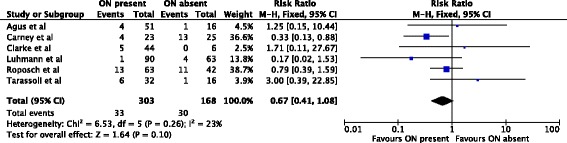
Forest plot for the development of osteonecrosis of grade II or worse at two or more years following open or closed reduction. Including the data of six studies there were 471 hips analyzed. There was no statistically significant association between the ossific nucleus and the development of osteonecrosis

An analysis of osteonecrosis grades II–IV included six studies (471 hips) [1, 2, 4, 7, 8, 12]. 33/303 (11%) hips with an ossific nucleus developed osteonecrosis compared with 30/168 (18%) without an ossific nucleus (relative risk, 0.67; 95% confidence interval, 0.41-1.08) (Fig. 3).

**Fig. 4:**
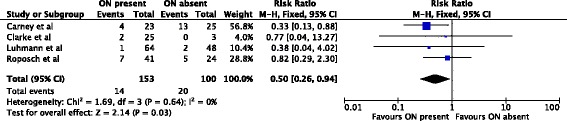
Forest plot for the development of osteonecrosis of any grade II or worse at two or more years following closed reduction. Including the data of four studies there were 253 hips analyzed. There was a statistically significant protective association between the ossific nucleus and the development of osteonecrosis

**Fig. 5:**
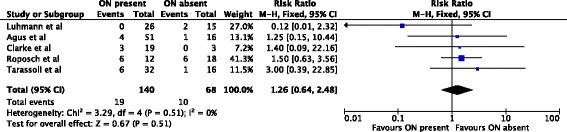
Forest plot for the development of osteonecrosis of grade II or worse at two or more years following open reduction. Including the data of five studies there were 208 hips analyzed. There was no statistically significant association between the ossific nucleus and the development of osteonecrosis

**Fig. 6:**
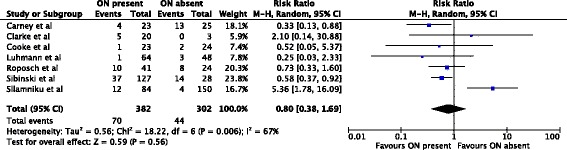
Forest plot for the development of osteonecrosis of any grade at two or more years following closed reduction. Including the data of seven studies, 684 hips were analyzed. There was no statistically significant association between the ossific nucleus and the development of osteonecrosis

**Fig. 7:**
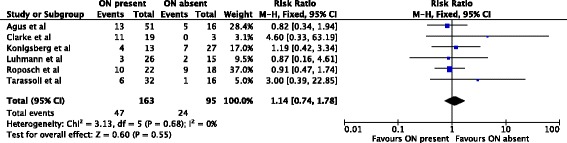
Forest plot for the development of osteonecrosis of any grade at two or more years following open reduction. Including the data of six studies, 258 hips were analyzed. There was no statistically significant association between the ossific nucleus and the development of osteonecrosis

In 253 hips treated by closed reduction only, the ossific nucleus reduced the risk for osteonecrosis of grades II-IV by 50%. 14/153 (9%) hips with an ossific nucleus developed osteonecrosis compared with 20/100 (20%) in the group without an ossific nucleus (relative risk, 0.50; 95% confidence interval, 0.26-0.94) (Fig. 4). The reminder of the subgroup analyses did not show significant effects (Figs. 5, 6 and 7).

### Publication bias

**Fig. 8:**
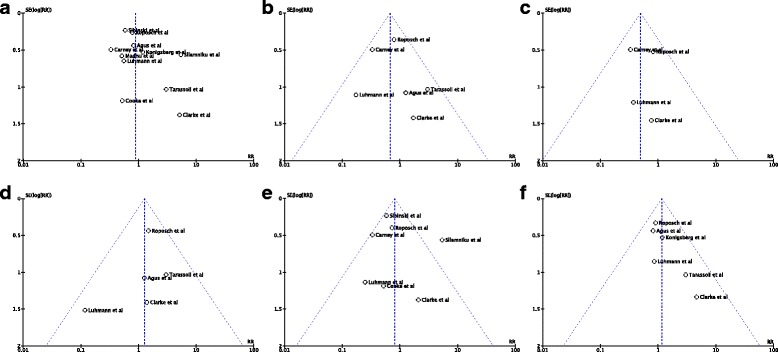
Begg’s funnel plots for assessment of publication bias. Each circle demonstrates study included in meta-analysis. 95% CI lines shown in fixed effects models. **a** Osteonecrosis of any grade at two or more years following open or closed reduction. **b** Osteonecrosis of any grade II or worse at two or more years following open or closed reduction. **c** Osteonecrosis of any grade II or worse at two or more years following closed reduction. **d** Osteonecrosis of any grade II or worse at two or more years following open reduction. **e** Osteonecrosis of any grade at two or more years following closed reduction. **f** Osteonecrosis of any grade at two or more years following open reduction

The generated Begg’s funnel plots showed points that were evenly distributed and symmetrical (Fig. 8). This shows that there is minimal publication bias and the results of this meta-analysis are credible.

## Discussion

Studies about the role of the femoral head ossific nucleus in preventing osteonecrosis following treatment of DDH reported conflicting results, but a 2009 meta-analysis concluded that there was no such effect unless only osteonecrosis grades II-IV were considered [9]. The meta-analysis, limited due to the small number of studies available at that time, identified a need for an update as further research emerges. We observed a trend in recent years towards delaying treatment of DDH in the absence of an ossified nucleus [1, 3, 21]. Numerous new publications on this topic encouraged us to update the 2009 meta-analysis in the hope to find robust conclusions.

With 11 studies or 1021 infants, this updated meta-analysis included twice as many studies and three times as many infants as the one published previously [9]. The primary outcome, development of osteonecrosis of any grade, remained similar with a relative risk of 0.75 (95% confidence interval, 0.46–1.21) in the 2009 study, compared with 0.87 (0.55–1.38) in the present study. We used a random effects model to calculate the primary outcome, as the I-squared value was greater than 50. However, this resulted in a Tau-squared value of 0.27 which shows low heterogeneity between studies. We also performed a fixed effects calculation on the same data set in order to compare results, as the random effect model assumes an average effect of the ossific nucleus on osteonecrosis across all studies. This showed similar results (Additional file 1). For the subgroup analysis we used a fixed effect model as low heterogeneity between studies was identified in the primary outcome meta-analysis, the number of studies was small and we felt it would be impossible to estimate the tau-squared value with any precision. We suggest this is confirmatory evidence that the ossified ossific nucleus does not play a role in the prevention of osteonecrosis – there does not seem to be a causal relationship.

The 2009 study showed a 60% reduction in the relative risk for developing osteonecrosis grade II or worse if the nucleus was ossified at the time of hip reduction (relative risk, 0.43, 95% confidence interval, 0.20–0.90) [9]. However, this subgroup analysis was based on only four studies [9]. We were able to update this subgroup analysis by including two additional studies [4, 8], which were of high quality based on the GRADE criteria. With an increased sample size from 318 to 471 infants, the updated result was no longer statistically significant – there was no effect of the ossified nucleus on the development of osteonecrosis grades II or worse. While the upper bound of the 95% confidence interval for this analysis was 1.08, we suggest that with a point estimate of 0.67 and the relatively high quality of the additional studies, this result is definitely negative.

The 2009 meta-analysis [9] also showed that the presence of the ossific nucleus reduced the probability of grade I–IV osteonecrosis by 60% (relative risk = 0.41, 95% confidence interval, 0.18–0.91) after closed reduction. This conclusion was based on three available studies or 183 infants. The updated analysis of closed reductions included seven studies or 684 patients showed no longer a protective effect with a relative risk of 0.80 (95% confidence interval, 0.38–1.69).

By contrast, infants treated with closed reductions (253 hips from four studies) [1, 2, 7, 9] demonstrated a 50% reduction in risk of developing osteonecrosis grade II-IV (relative risk, 0.50; 95% confidence interval, 0.26–0.94). A possible explanation for this finding is that a closed reduction performed on an ossified nucleus represents a lesser insult to a less plastic epiphysis [2, 3]. It may be that during an open reduction the decreased plasticity of the ossified nucleus is more than offset by the trauma associated with surgery and possible disruption of the capsular blood supply.

We noticed inconsistencies in the reported prevalence of osteonecrosis, which ranged from 6 to 48%. Those studies that reported a prevalence of 6 to 7% [7, 14, 20] included predominately cases of closed reductions. Further, one study [7] excluded patients with incomplete notes or imaging, and another study [20] excluded patients below the age of two months and those over 20 month. Selection bias could possibly account for the low prevalence of osteonecrosis in these studies. Measurement bias is another possible explanation for the wide range in prevalence estimates. Only seven studies (50%) stated the number of outcome assessors [2, 8, 12–14, 19, 20] and only two studies (14%) [8, 13] evaluated outcomes blinded and included inter-rater reliability studies. Attrition bias was inherent to the study of Pospischill et al. [13], which excluded 21 patients (21%) due to length of follow-up being less than three years.

All included studies identified the age at reduction as a potential confounder in the relationship between osteonecrosis and an ossific nucleus; however, only ten studies (72%) [1–3, 5–8, 12–14] provided supportive statistical analyses. Other confounders have been reported: Carney et al. [1] found an increased risk of osteonecrosis when an adductor tenotomy was omitted as part of a closed reduction (*p* = 0.0066). In the study of Pospischill et al. [13] osteonecrosis was more likely (*p* = 0.003) when a concomitant osteotomy was done. Only two studies (14%) performed multivariate analyses [8, 13].

Our study has limitations. In four studies (29%) the presence of the ossific nucleus was determined solely or in select cases by ultrasound. Because previous research has demonstrated that in 93% of cases an ossific nucleus that is visible on ultrasound is also visible on radiographs [22] we included these papers in our study. Three studies (22%) [1, 12, 19] had a follow up of only two years. It could be argued that this might underestimate the prevalence of osteonecrosis; however, two years has been used as an outcome in previous research [9] and is perhaps acceptable for making reasonable inferences. There was variation in the radiological classifications used for grading the severity of osteonecrosis in different studies. Thirteen studies (93%) used Bucholz and Ogden [1, 3, 6–8, 13, 14, 16] or Kalamchi and MacEwen [2, 4, 5, 12, 20] which are interchangeable. Madhu et al. [19] graded osteonecrosis according to Salter et al. [23] and we thus could include their data only when modeling the effects of the ossific nucleus on osteonecrosis of grades I–IV.

## Conclusion

Based on current evidence there does not appear to be a protective effect of the ossific nucleus on the development of osteonecrosis. In contrast to the previous meta-analysis, this update demonstrates that this remains the case irrespective of the grade of osteonecrosis considered relevant. This updated meta-analysis is based on twice as many studies with a higher quality of evidence and therefore provides more robust conclusions. There does appear to be a protective effect against the development of grade II-IV osteonecrosis when closed reductions are performed. Caution must be exercised as the number of studies on which this conclusion is based is small and the clinical implication is unclear since the likelihood of successful closed reduction reduces with time. We therefore conclude that the practice of delaying open reduction until the appearance of the femoral ossific nucleus is not currently supported by the literature. However this meta-analysis is based on exclusively retrospective studies and the quality of evidence remains moderate. It is the opinion of these authors that the question of whether delaying the treatment of a dislocated hip in the absence of the ossific nucleus can mitigate the risk for osteonecrosis would be best answered by a randomized clinical trial.

## Additional file

Additional file 1: Forest plots showing a fixed or random effects models equivalent to Figs. 2, 3, 4, 5, 6 and 7. a. Data used in Fig. 2 analysed using a fixed effects model. b. Data used in Fig. 3 analyzed using a random effects model. c. Data used in Fig. 4 analyzed using a random effects model. d. Data used in Fig. 5 analyzed using a random effects model. e. Data used in Fig. 6 analyzed using a random effects model. f. Data used in Fig. 7 analyzed using a random effects model. (DOCX 79 kb)

## Abbreviations

DDH: Developmental Dysplasia of the Hip
GRADE: Grading of Recommendations Assessment, Development and Evaluation
MeSH: Medical Subject Headings
PRISMA: Preferred Reporting Items for Systematic Reviews and Meta-Analyses
