# Supplementary figures and images for: Association between the ossific nucleus and osteonecrosis in treating developmental dysplasia of the Hip: updated meta-analysis
Source: BMC Musculoskelet Disord. 2017 Apr 20;18:165. doi: 10.1186/s12891-017-1468-6 (PMC5397826; doi:10.1186/s12891-017-1468-6)

**a**


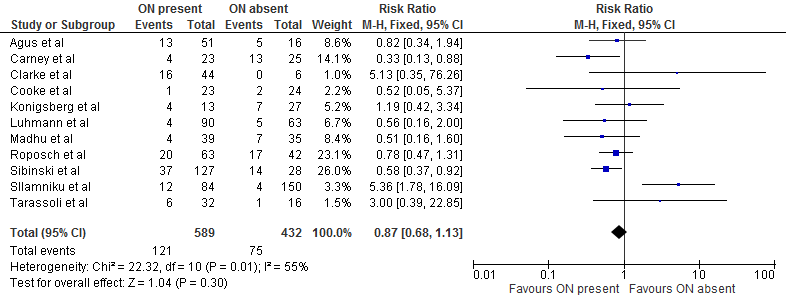


**b**


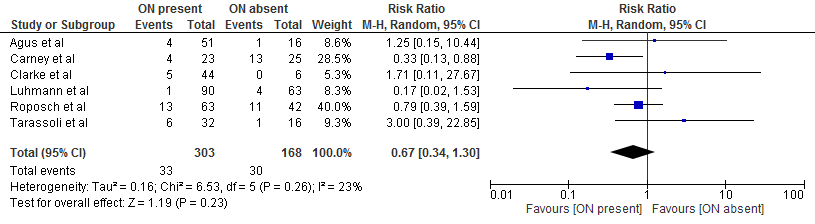


**c**


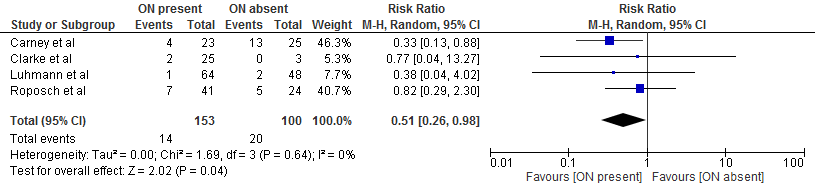


**d**


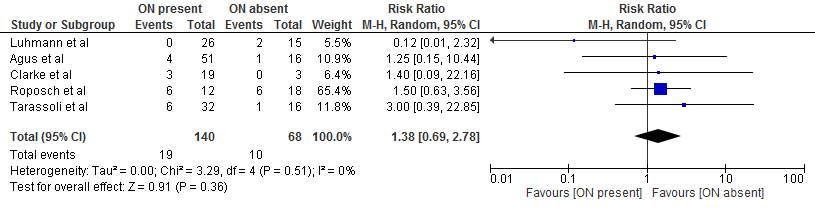


**e**


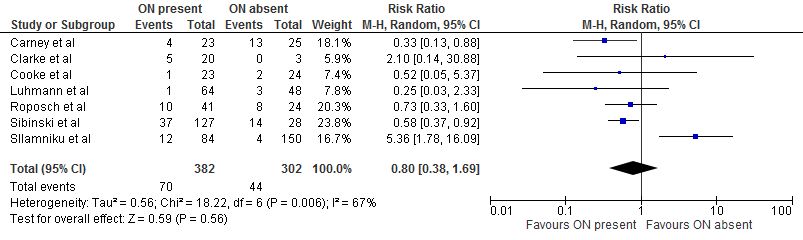


**f**


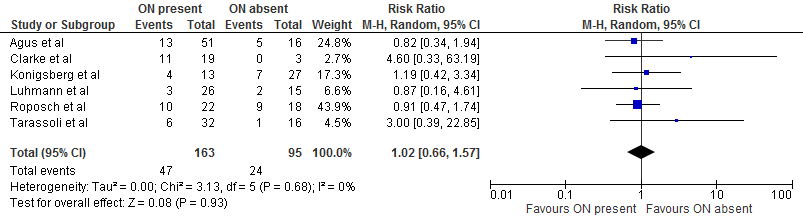

Supplement: Additional file 1: — Forest plots showing a fixed or random effects models equivalent to Figs. 2, 3, 4, 5, 6 and 7. a. Data used in Fig. 2 analysed using a fixed effects model. b. Data used in Fig. 3 analyzed using a random effects model. c. Data used in Fig. 4 analyzed using a random effects model. d. Data used in Fig. 5 analyzed using a random effects model. e. Data used in Fig. 6 analyzed using a random effects model. f. Data used in Fig. 7 analyzed using a random effects model. (DOCX 79 kb) [file 12891_2017_1468_MOESM1_ESM.docx]
